# A Retrieval System for Patients with Avoidable Blindness Due to Diabetic Retinopathy who do not Present for Ophthalmic Assessment in Oman

**DOI:** 10.4103/0974-9233.80694

**Published:** 2011

**Authors:** Rajiv Khandekar, Jawad Al Lawati, Nabil Barakat

**Affiliations:** Department of Non-communicable Disease Control, Eye and Ear Health Care, Ministry of Health, Oman

**Keywords:** Defaulter, Diabetes Mellitus, Diabetic Retinopathy

## Abstract

**Background:**

Many patients with diabetes do not present for eye examinations, foregoing the recommended management for diabetic eye care. Proactive steps are being taken in Oman to retrieve defaulters (patients who do not present or “no-show”) with Sight Threatening Diabetic Retinopathy (STDR). We present the outcomes of the defaulter retrieval system in five regions of Oman in 2009.

**Materials and Methods:**

Ophthalmologists examine eyes periodically, family physicians focus on primary prevention of Diabetic Retinopathy (DR) and medical retina specialists manage DR in Oman. A person with proliferative stage of DR (PDR) and/or Diabetic Macular Edema (DME) in either eye is considered as STDR and is registered at regional hospitals. The eye care staff identify the defaulters and the hospital staff help them retrieve the defaulters. The reminder of reappointment is sent using the text messages on telephone. The glycemic control of STDR cases was also noted in Nizwa Hospital.

**Results:**

We registered 654 STDR cases, of which 494 (75%) were defaulters. Lack of awareness, transport, absence of a decision maker, and fear of laser treatment were the main causes for defaulting. We successfully retrieved 328 (66.4%) defaulters. The retrieval rates among male and female patients were 51.2% and 82%, respectively. The retrieval varied by region. In Nizwa hospital, 114 of 131 STDR cases (85%) had poor glycemic control.

**Conclusion:**

Defaulter retrieval system could help healthcare providers to identify and motivate patients with STDR towards better compliance. Primary prevention measures among STDR cases were poor and need further focus.

## INTRODUCTION

If the mountain does not come to Mohammed, Mohammed will go to the mountain.’^1^ This proverb was well used by public health personnel during mass public immunizations and vaccinations for communicable diseases.^2^ Those who do not present for vaccination on the scheduled date are called defaulters and the health staff/village workers approach parents to motivate them to cooperate in vaccinating their children. A similar approach could be adopted for the control of chronic and noncommunicable diseases, such as diabetes. Such a proactive approach could improve the coverage of screenings and standard treatment.

Diabetes is an epidemic in Gulf countries.^3^ The preliminary report of World Health Survey suggests that the prevalence of adult diabetes was 17% in 2010 in Oman (Personal communication on November 1, 2010, Dr Asya Al Riyami, Director of Research, Ministry of Health, Oman). Diabetic retinopathy (DR) is a micro-vascular complication of diabetes and develops in more than 75% of individuals with a 20-year duration of type II diabetes and in all persons with type I diabetes.^4^ Diabetics have 20 times higher risk of developing visual disabilities than the healthy population.^5^ Fortunately, visual disabilities can be prevented or delayed by early detection and timely management of DR.^6^ Therefore, the World Health Organization (WHO) prioritized DR in its VISION 2020 initiative and recommended member countries to adopt a public health approach for DR.^7^ Due to low rates of presentation for ophthalmic assessment and low compliance, a proactive approach form healthcare workers is required.^8^ Tracking all diabetics is not possible. Hence, individuals with Sight Threatening DR (STDR) must be identified, managed, and judiciously followed up to delay or avoid visual morbidity.

Oman, a member country of Gulf Council and WHO Eastern Mediterranean Region, has introduced annual eye screening of all registered diabetics since 1996.^9^ By the end of 2009, 61,583 diabetics were registered.^10^ Although these patients are counseled to have regular eye examinations, not all of them present for ophthalmic evaluation as advised. Diabetics classified as not having DR on their first or second ophthalmic assessment are much or more likely to be noncompliant. Tracking all of them is not logistically possible. Therefore, the staff of eye healthcare and diabetes control program focus on the most vulnerable group, ie, patients with STDR.^11^ STDR is defined as a person having proliferative diabetic retinopathy (PDR) and/or diabetic macular edema (DME) due to DR in either eye.^12^ To identify these individuals, a registry for STDR was introduced at selected eye units where both screening and laser treatment facilities were available. If a patient with STDR did not present for the scheduled appointment, he/she was labeled as a defaulter. Since 2007, attempts were made to retrieve these defaulters.

We present our experience of the STDR registry and retrieval of defaulters in Oman. On the basis of this experience, we propose a model to plan better ophthalmic care of STDR cases and implement a defaulter retrieval system.

## MATERIALS AND METHODS

This operational research was a part of monitoring the health program in the country. The national eye health care committee gave the consent for this study. The study was conducted in 2010. We reviewed the STDR register data [Table 1] for 2009 from five of nine regional hospitals, namely Nizwa, Ibra, Sur, Sohar, and Ibri. The ophthalmologists and nursing staff of ophthalmic units were the field investigators for the study. An ophthalmologist with subspecialty in medical retina conducted a training workshop for them.

**Table 1:** Sight threatening diabetic retinopathy register

Name of the Institute: ________________________							
Region: ______________________							

Diabetic register^#^	Patient)s sticker	Sheikh)s name	Contact Phone^#^	Alternate contact Phone^#^	Date appointment	Defaulted (Yes/No)	Rescheduled appointment*


* Please include only cases with severe preproliferative diabetic retinopathy above macula, proliferative diabetic retinopathy, and diabetic maculopathy,

**Rescheduled appointment is given by the record section of the hospital to defaulters

For detailed retinal examination by a dilated pupil, one drop of 1% tropicamide was instilled in each eye. If dilation was not adequate, the eye drop was repeated after 30 minutes. The individuals were observed for one hour after dilation to monitor intraocular pressure and manage any increases in intraocular pressure. The ophthalmologists used a slit lamp biomicroscope (Topcon Corp., Tokyo, Japan) and +90 D lens for funduscopy. They documented the retinal images of each eye with digital fundus cameras (Carl Ziess, Jena, Germany, and Kowa Co, Ltd, Nagoya, Japan). DR grading was based on the modified Early Treatment Diabetic Retinopathy Study (EDTRS) classification.^12^

All STDR registered cases were included in our study. The information about gender, location by region, and telephone number was collected for further communication from computerized health records. Individuals with STDR who failed to present for their scheduled appointment were labeled as ‘Defaulters Type I’. If a STDR patient was advised to undergo laser treatment and did not present on the scheduled date for the laser treatment, he/she was labeled as ‘Defaulter type II’. The staff of eye department and medical records section of the hospital communicated with these individuals. In two regions (Nizwa and Ibra), the wilayat (district) administrators were also involved in retrieving defaulters. They contacted the patient or his/her near relative within three days after the missed appointment. They tried to find the cause of the ‘no show’ and, if possible, eliminated the barrier with the help of relatives and hospital administration. The defaulters were counseled again, provided transport in case of nonavailability of transport for returning home. We studied the process of defaulter retrieval and its success.

The report of glycemic control on the day of reappointment after defaulting was noted in Nizwa hospital to determine the status of primary prevention of STDR.

We also conducted interviews of the health staff of the study sites to determine the underlying causes of ‘no show’ based on their telephone interview with the patients.

Data were computed using Microsoft Excel^®^ (Microsoft Corp, Redmond, WA, USA). Univariate analysis was performed with Statistical Package for Social Studies (SPSS version 11, SPSS Inc., Chicago, IL, USA). The frequencies and percentage were calculated for different variables. The mean and standard deviation were calculated for blood sugar level of all STDR cases reported at Nizwa hospital.

Periodic visits to hospital and meetings with field investigators enabled us to standardize the registry and collect all data relevant to this study.

## RESULT

In 2009, a total of 9,517 diabetics (including newly diagnosed individuals) were registered in the study area. From this cohort, 654 [6.87% (95% confidence interval, 6.36–7.38)] were registered with STDR. A total of 160 individuals (25%) with STDR reported for further evaluation and management on the given appointment date. The remaining 494 individuals (250 male and 244 female) were defaulters. There were 345 (53%) type I defaulters and 149 (22.8%) type II defaulters. Using a proactive approach, we retrieved 328 defaulters (66.4%; 128 male and 200 female). We retrieved 150 cases in Nizwa, 12 cases in Ibra, 25 cases in Sur, 92 cases in Sohar, and 49 cases in Ibri. Among retrieved defaulters, 37 (25%) agreed to undergo laser treatment.

Defaulting patients stated that the main barriers to presenting for an appointment were lack of transport, lack of awareness regarding the risk of blindness, fear of laser treatment, and absence/reluctance of the decision maker in the family for the proposed management.

**Figure 1:**
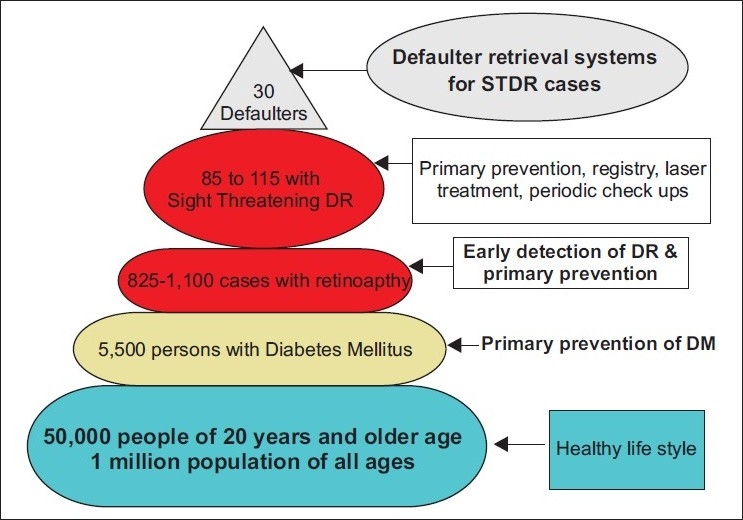
Projections of cases with Sight Threatening Diabetic Retinopathy and defaulters

Of the 131 cases with STDR at Nizwa Hospital of Dhakhiliya region of Oman (the only hospital where such data were collected), the mean blood sugar level was 13.2±5.25 mmol/dl. The fasting blood sugar level was more than 7 mmol/dl in 114 (87%) of STDR cases [Figure 1].

**Figure 2:**
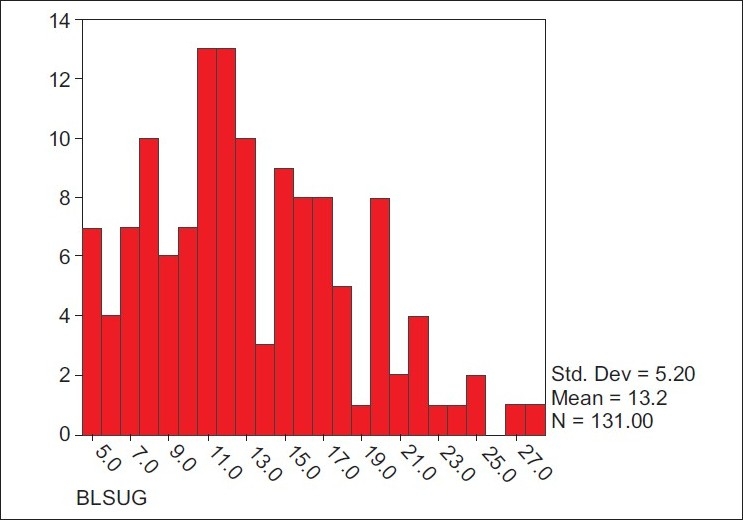
Blood sugar levels among individuals with Sight Threatening Diabetic Retinopathy registered at Nizwa Wilayat of Oman

A schematic model is presented to estimate the number of diabetics, diabetic retinopathy cases, and STDR and defaulters in a population of one million [Figure 2].

## DISCUSSION

The public health approach to address diabetes and its complication is challenging. This burden will increase as the number of adults diabetics worldwide increases to 439 million by 2030.^13^ In Eastern Mediterranean countries, the prevalence of diabetes was in the range of 7.2-20.5%.^14^ In Bahrain, the prevalence of diabetes was reported to be 30% among adult population.^15^ Thus, focusing on all individuals with diabetes, although desirable, may not be possible even in resourceful Gulf Cooperation Council (GCC) countries, such as Bahrain.

Due to longer survival rates of diabetics over time, the incidence of DR is further increasing. In the US, diabetes has been forecasted to increase from 5.5 million in 2005 to 16.0 million by 2050.^16^ In UAE, Qatar, and Saudi Arabia, the prevalence of DR is 19%, 23.5%, and 30%, respectively.^17^–^19^ In Oman, the prevalence of DR was 14.5% and 42.4% in two studies.^20^,^21^ Hence, most of the GCC countries are already facing a severe public health burden of DR. The global VISION 2020 initiatives in these countries face an uphill challenge in reducing visual impairment due to diabetes.

In the US, STDR is projected to increase from 1.2 million in 2005 to 3.4 million by 2050.^16^ A study in Britain suggested that the progression rate of developing STDR in cases with background DR is 5%, and with the preproliferative stage of DR, it is 15% in the first year after diagnosis.^22^ In our study, we found a 7% prevalence of STDR among registered diabetics. In view of poor primary prevention of diabetes, the rate of STDR could increase in the coming years. Primary prevention of diabetes, regular eye examinations, and timely management have shown encouraging results in avoiding or delaying visual disabilities.^23^ Unfortunately, poor compliance by diabetics is a hindrance in implementing these strategies. Identifying STDR cases and focusing on them could be a proactive step.

The STDR registry and defaulter retrieval system in Oman have enabled the national prevention of blindness program to keep track of this high-risk group. Tracking a case that failed to report for follow-up was a major challenge. Routinely, health administrators use different modes of communication in Oman such as short messaging system (SMS) and telephone reminders about appointments. For STDR defaulters, we used these communication channels and also involved primary health staff of the village. Although, these are resource-intensive initiatives, other Gulf countries with adequate resources can adopt these to improve eye care in STDR cases.

The overall rate of timely follow-up among STDR cases was very low in our study. In the US, a study reported that 79% of female diabetics were compliant for eye examinations.^24^ In Switzerland, medical advice for regular check-ups was followed by 50% of diabetics.^25^ In Spain, 44% of patients did not comply for regular blood test for monitoring diabetes.^26^ Our cohort being with very high-risk of visual disability, we expected a higher compliance rate. However, 75% were defaulters in this group, which is disconcerting and warrants implementation of a defaulter retrieval system.

We found a lack of awareness and transportation were hindrances to presenting for an appointment. The main cause of noncompliance in the US was financial and lack of awareness about the need for an eye examination among female diabetics.^24^ Bieschoff reported that 25% of diabetics were unaware of the risk of visual disability.^25^ Thus, barriers to compliance seem to differ in different countries, and hence efforts to overcome these barriers should consider such variations as well.

Our retriever system successfully recruited approximately two-thirds of defaulters. This is an encouraging outcome. Addressing the barriers will improve eye care for diabetics in Oman. The system retrieved more females compared to males. Perhaps, female patients were facing barriers that could be addressed through personal reminders and rescheduling the appointments based on their convenience. The significant proportion of type II defaulters in Oman is a matter of concern. The underlying causes of failure to present should be further investigated with a larger sample and addressed accordingly.

Regional variation in a defaulter retrieval suggested that cooperation of healthcare staff, administrators, and patients in improving the eye care system varied by region. Even methods used for retrieval at different sites varied. Hence, it is difficult to determine which mode of retrieval was better.

Public health education for judicious glycemic control among STDR cases is urgently needed in Oman. This is also reflected the attitude of diabetics with advanced stages of retinopathy towards primary prevention. Even in an industrialized nation such as Germany, the compliance for laser treatment was poor.^27^ Any public health program directed at early detection and timely treatment should, therefore, be complemented by patient compliance for primary prevention and periodic eye assessment.

The defaulter retrieval system to reduce visual disabilities due to STDR in Oman exists but more efforts and expansion are needed to cover all regions. The cost-effectiveness of such an initiative should be studied to further strengthen our case for promoting a retrieval system. The stress on primary prevention must be for all cases of DR, with special attention for those with STDR. The healthcare staff involved in diabetes control program could contribute more actively in the primary prevention and advocacy for regular eye examinations. These steps will help in reducing avoidable blindness caused by diabetes.
